# Editorial: Antimicrobial resistance: tracking and tackling in the food chain

**DOI:** 10.3389/fmicb.2026.1769277

**Published:** 2026-01-20

**Authors:** Teresa Semedo-Lemsaddek, Byeonghwa Jeon, Narjol González-Escalona, Marta Laranjo

**Affiliations:** 1 Centre for Interdisciplinary Research in Animal Health (CIISA), Faculty of Veterinary Medicine, University of Lisbon, Lisbon, Portugal; 2 Associate Laboratory for Animal and Veterinary Sciences (AL4AnimalS), Lisbon, Portugal; 3 Biosystems and Integrative Sciences Institute (BioISI), Faculty of Sciences, University of Lisbon, Lisbon, Portugal; 4 Environmental Health Sciences, School of Public Health, University of Minnesota, St. Paul, MN, United States; 5 Genomics Development and Applications Branch, Division of Food Safety Genomics, Office of Applied Microbiology and Technology (OAMT), Office of Laboratory Operations and Applied Science (OLOAS), Human Foods Program (HFP), U.S. Food and Drug Administration, Silver Spring, MD, United States; 6 MED-Mediterranean Institute for Agriculture, Environment and Development & CHANGE-Global Change and Sustainability Institute, Departamento de Medicina Veterinária, Escola de Ciências e Tecnologia, Universidade de Évora, Évora, Portugal

**Keywords:** AMR, food safety, foodborne pathogen detection in foods, horizontal gene transfer (HGT), mitigate antimicrobial resistance

Antimicrobial resistance (AMR) poses a continuous threat to human health, animal welfare and the resilience of food systems. The food chain constitutes a dynamic network where microbes, resistance genes and selective pressures move between farms, processing environments, retail settings and consumers. Therefore, addressing AMR requires evidence that links these compartments, rigorous genomic and ecological analysis, and actionable interventions that reduce selection pressure and block transmission. The Research Topic *Antimicrobial Resistance: Tracking and Tackling in the Food Chain* assembles 10 studies that together deliver such evidence, combining field surveillance, genomic epidemiology, resistome profiling and exploratory control strategies. Collectively, these contributions sharpen our understanding of where risks are concentrated, how resistance circulates, and which interventions merit priority.

The Research Topic highlights three interrelated messages. First, diverse foodborne pathogens and commensal taxa frequently carry multiple resistance determinants, including genes located on mobile elements, which facilitates their spread. Second, processing environments and waste streams are important reservoirs of resistance and potential dissemination pathways to the wider environment. Third, a One Health approach that integrates animal, human and environmental surveillance is essential to reveal transmission routes and to develop effective mitigation strategies.

Market and consumer surveillance immediately before consumption reveals persistent risks. In the article by Letuka et al., ready-to-eat street foods sampled in Mangaung, South Africa, contained widespread *Staphylococcus* species and a notable proportion of multidrug resistant *S. aureus* isolates that harbor numerous virulence genes. These findings stress that informal retail settings can sustain both pathogenic potential and AMR and therefore deserve targeted monitoring and community-engaged risk-reduction strategies.

Production-stage surveillance provides complementary insights. In the article by Liu X. et al., over 500 *Escherichia coli* isolates from large-scale broiler farms in Shandong Province were profiled across production stages. Multidrug resistance was pervasive, peaked at a specific growth stage, and correlated with the use of specific antimicrobials, such as doxycycline. This study reinforces that antimicrobial stewardship policies and farm-level management must be time- and context-sensitive to effectively interrupt selection pressures.

Moreover, animal health contexts can show contrasting local realities. In the article by Moawad et al., *S. aureus* from small ruminant mastitis in Sardinia displayed considerable genetic diversity but remained largely susceptible to commonly used mastitis antimicrobials. This result serves as an important reminder that AMR is heterogeneous: pockets of low resistance exist and should be actively preserved through responsible treatment protocols and biosecurity.

Processing facilities and side-streams emerge as significant reservoirs of genetic resistance. Reiche et al. used targeted hybrid capture sequencing to map resistomes in salmon and broiler processing plants and found high densities of AMR genes in wastewater and sludge, including determinants associated with multidrug and beta-lactam resistance. Though phenotypically resistant isolates in side-stream materials were relatively infrequent, the genomic abundance of AMR genes in effluents indicates a tangible risk of environmental recycling and re-introduction to production systems.

Retail-ready products likewise carry detectable resistance. In the article by Thoenen et al., a systematic sampling of Swiss ready-to-eat meat identified multiple antibiotic-resistant organisms, many multidrug resistant, and resistance genes spanning numerous classes. The detection of metal resistance genes highlights co-selective pressures that are independent of antibiotic use and that can sustain resistance within the food chain.

These empirical studies are complemented by research that probes the broader resistome and mechanisms of gene flow. Zhao et al. combined high-throughput qPCR and 16S profiling in raw milk from northwest Xinjiang to show that ARG distributions are shaped by microbiota composition, physicochemical parameters and mobile genetic elements. Liu Q. et al. report extensive contamination of raw milk in Jilin with *S. aureus*, including MRSA and linezolid resistance genes, with genomic signals of human-livestock transmission-evidence that raw milk is a critical interface for cross-host exchange. Zhu et al. performed comparative genomics of *Clostridium perfringens* from animal-derived foods and human sources, revealing an open pan-genome, frequent prophage carriage and shared sequence types, which together point to cross-sectoral connectivity.

Mechanistic studies deepen our understanding of horizontal gene transfer. Stein et al. used exogenous plasmid capture from retail sprouts to recover conjugative tetracycline-resistance plasmids, demonstrating that minimally processed produce can act as a donor pool for mobile resistance elements. This finding underscores the need to consider plant-based foods in AMR surveillance frameworks.

Finally, the Research Topic points toward pragmatic control options. Zhang et al. demonstrate that LysP70, an endolysin from a *Listeria* phage, effectively lyses *Listeria monocytogenes* and disrupts biofilms in milk, illustrating how targeted bacteriophage-derived agents can complement reduced antibiotic use and strengthen food safety controls.

Taken together, the studies in this Research Topic deliver a rigorous, evidence-based portrait of AMR in food systems. They identify where surveillance should be intensified, which sectors warrant immediate stewardship and management changes, and where innovations, such as phage-derived antimicrobials, offer promise. Key priorities that emerge are harmonized, multisectoral surveillance that links genomics with phenotypic testing; interventions that reduce selection pressure at critical production stages; improved treatment and management of processing effluents; and inclusion of non-traditional vectors and indicators, such as produce-associated plasmids and metal-resistance markers, in monitoring programs.

We thank all authors and reviewers for their timely contributions. The works included in the current Research Topic advance a One Health agenda based in rigorous data and practical applicability. Continued collaboration among microbiologists, veterinary doctors, food safety practitioners, environmental scientists, and policymakers will be essential to translate these findings into sustained reductions in AMR risk across the different food value chains.

